# {*N*′-[(*E*)-1-(5-Bromo-2-oxidophen­yl)ethyl­idene-κ*O*]-4-methyl­benzohydrazidato-κ^2^ *N*′,*O*}(pyridine-κ*N*)nickel(II)

**DOI:** 10.1107/S1600536811029114

**Published:** 2011-07-23

**Authors:** Chang-Zheng Zheng, Liang Wang, Juan Liu, Yu-Jie Wang

**Affiliations:** a College of Environment and Chemical Engineering, Xi’an Polytechnic University, 710048 Xi’an, Shaanxi, People’s Republic of China

## Abstract

The central Ni^II^ atom in the title complex, [Ni(C_16_H_13_BrN_2_O_2_)(C_5_H_5_N)], is in a square-planar *trans*-N_2_O_2_ environment defined by the NO_2_ donor atoms of the tridentate hydrazone ligand and the monodentate pyridine ligand. The pyridine mol­ecule forms a dihedral angle of 9.99 (11)° with the least-squares plane through the NiN_2_O_2_ atoms.

## Related literature

For the biological and coordination properties of aroylhydra­zones, see: Ali *et al.* (2004 ▶); Carcelli *et al.* (1995 ▶); Cheng *et al.* (1996 ▶); Desai *et al.* (2001 ▶); El-Masry *et al.* (2000 ▶); Singh & Dash (1988 ▶); Zheng *et al.* (2008 ▶).
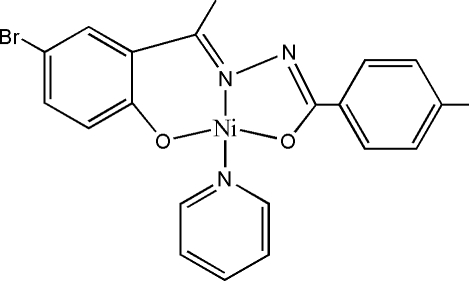


## Experimental

### 

#### Crystal data

[Ni(C_16_H_13_BrN_2_O_2_)(C_5_H_5_N)]
*M* *_r_* = 483.00
Monoclinic, 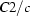

*a* = 32.376 (18) Å
*b* = 6.145 (4) Å
*c* = 22.752 (13) Å
β = 122.063 (8)°
*V* = 3836 (4) Å^3^
*Z* = 8
Mo *K*α radiation
μ = 3.12 mm^−1^
*T* = 298 K
0.21 × 0.16 × 0.12 mm

#### Data collection

Bruker SMART CCD area-detector diffractometer
Absorption correction: multi-scan (*SADABS*; Sheldrick, 1996 ▶) *T*_min_ = 0.561, *T*_max_ = 0.706
9451 measured reflections
3403 independent reflections
2415 reflections with *I* > 2σ(*I*)
*R*_int_ = 0.044

#### Refinement

*R*[*F* ^2^ > 2σ(*F* ^2^)] = 0.038
*wR*(*F* ^2^) = 0.093
*S* = 1.03
3403 reflections
253 parameters
H-atom parameters constrained
Δρ_max_ = 0.40 e Å^−3^
Δρ_min_ = −0.38 e Å^−3^

### 

Data collection: *SMART* (Bruker, 1996 ▶); cell refinement: *SAINT* (Bruker, 1996 ▶); data reduction: *SAINT*; program(s) used to solve structure: *SHELXS97* (Sheldrick, 2008 ▶); program(s) used to refine structure: *SHELXL97* (Sheldrick, 2008 ▶); molecular graphics: *SHELXTL* (Sheldrick, 2008 ▶); software used to prepare material for publication: *SHELXTL*.

## Supplementary Material

Crystal structure: contains datablock(s) I, global. DOI: 10.1107/S1600536811029114/tk2766sup1.cif

Structure factors: contains datablock(s) I. DOI: 10.1107/S1600536811029114/tk2766Isup2.hkl

Supplementary material file. DOI: 10.1107/S1600536811029114/tk2766Isup3.cdx

Additional supplementary materials:  crystallographic information; 3D view; checkCIF report

## Figures and Tables

**Table 1:** Selected bond lengths (Å)

Ni1—O1	1.794 (3)
Ni1—O2	1.826 (3)
Ni1—N2	1.835 (3)
Ni1—N3	1.941 (3)

**Fig. 1.**
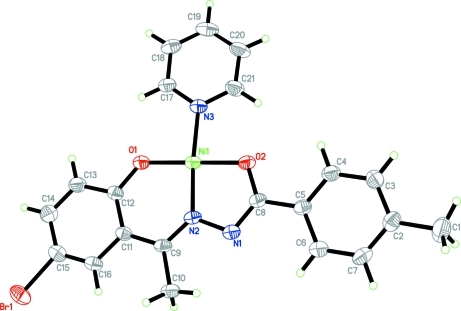
The molecular structure of the title compound, showing displacement ellipsoids drawn at the 30% probability level. H atoms are presented as a small spheres of arbitrary radius.

## supplementary crystallographic information

### Comment

Hydrazones are an important class of Schiff bases compounds which has attracted much attention because of their biological activities (Carcelli *et al.*, 1995) such as antimicrobial, antifungal, antitumor and as herbicides (El-Masry *et al.*, 2000; Singh & Dash, 1988; Desai *et al.*, 2001), and strong tendency to chelate to transition metals (Ali *et al.*, 2004; Cheng *et al.*, 1996). As an extension of our work on the structural characterization of aroylhydrazone derivatives (Zheng *et al.*, 2008), the title compound was synthesized and its crystal structure is reported here.

The coordination polyhedron about the nickel ion in the title complex is essentially planar, Fig. 1. The coordination environment of nickel is comprised of one pyridine ligand and one hydrazone ligand (two O atoms, one N atom) so that the central nickel atom is four-coordinated, Table 1.

### Experimental

*p*-Methyl ethylbenzoate (8.21 g, 0.05 mol) was dissolved in ethanol (50 ml) at room temperature and heated at 363 K, followed by the addition of hydrazine hydrate (3.00 g, 0.060 mol). Subsequently, the mixture was refluxed for 10 h, and then cooled to room temperature. The crystals were precipitated and collected by filtration. The product was recrystallized from ethanol and dried under reduced pressure to give 4-methylbenzohydrazide.

4-methylbenzohydrazide (3.75 g, 0.025 mol) was dissolved in ethanol (50 ml) at room temperature and heated at 363 K, followed by the addition of 5-bromo-2-hydroxyphenyl ethyl ketone (5.38 g, 0.025 mol). Subsequently, the mixture was refluxed for 9 h, and then cooled to room temperature. The crystals were precipitated and collected by filtration. The product was recrystallized from ethanol and dried under reduced pressure to give compound *N*'- [(*E*)-(5-Bromo-2-hydroxyphenyl)-(methyl)methylene]-4-methylbenzohydrazide.

A mixture of *N*'-[(*E*)-(5-Bromo-2-hydroxyphenyl)-(methyl)methylene] -4-methylbenzohydrazide (0.035 g, 0.10 mmol), NiCl_2_.6H_2_O (0.024 g, 0.10 mmol), pyridine (0.0079 g, 0.10 mmol), and H_2_O (5.00 ml), several drops of acetone was placed in a Parr Teflon-lined stainless steel vessel (25 ml). The vessel was sealed and heated at 393 K for 3 d. After the mixture was slowly cooled to room temperature, red crystals were obtained (yield 41%).

### Refinement

All H atoms were positioned geometrically and treated as riding on their parent atoms,with C—H(methyl) = 0.96 Å, C—H(aromatic) = 0.93 Å, and with *U*_iso_(H) =1.5*U*_eq_(C_methyl_) and 1.2*U*_eq_(C_aromatic_).

### Crystal data

**Table:**

[Ni(C_16_H_13_BrN_2_O_2_)(C_5_H_5_N)]	*F*(000) = 1952
*M**_r_* = 483.00	*D*_x_ = 1.673 Mg m^−^^3^
Monoclinic, *C*2/*c*	Mo *K*α radiation, λ = 0.71073 Å
Hall symbol: -C 2yc	Cell parameters from 2511 reflections
*a* = 32.376 (18) Å	θ = 3.2–23.3°
*b* = 6.145 (4) Å	µ = 3.12 mm^−^^1^
*c* = 22.752 (13) Å	*T* = 298 K
β = 122.063 (8)°	Block, red
*V* = 3836 (4) Å^3^	0.21 × 0.16 × 0.12 mm
*Z* = 8	

### Data collection

**Table:**

Bruker SMART CCD area-detector diffractometer	3403 independent reflections
Radiation source: fine-focus sealed tube	2415 reflections with *I* > 2σ(*I*)
graphite	*R*_int_ = 0.044
φ and ω scans	θ_max_ = 25.1°, θ_min_ = 1.5°
Absorption correction: multi-scan (*SADABS*; Sheldrick, 1996)	*h* = −31→38
*T*_min_ = 0.561, *T*_max_ = 0.706	*k* = −6→7
9451 measured reflections	*l* = −27→26

### Refinement

**Table:**

Refinement on *F*^2^	Secondary atom site location: difference Fourier map
Least-squares matrix: full	Hydrogen site location: inferred from neighbouring sites
*R*[*F*^2^ > 2σ(*F*^2^)] = 0.038	H-atom parameters constrained
*wR*(*F*^2^) = 0.093	*w* = 1/[σ^2^(*F*_o_^2^) + (0.0398*P*)^2^ + 0.067*P*] where *P* = (*F*_o_^2^ + 2*F*_c_^2^)/3
*S* = 1.03	(Δ/σ)_max_ < 0.001
3403 reflections	Δρ_max_ = 0.40 e Å^−^^3^
253 parameters	Δρ_min_ = −0.38 e Å^−^^3^
0 restraints	Extinction correction: *SHELXL97* (Sheldrick, 2008)
Primary atom site location: structure-invariant direct methods	Extinction coefficient: 0.0113 (15)

### Special details

**Table:**

Geometry. All e.s.d.'s (except the e.s.d. in the dihedral angle between two l.s. planes) are estimated using the full covariance matrix. The cell e.s.d.'s are taken into account individually in the estimation of e.s.d.'s in distances, angles and torsion angles; correlations between e.s.d.'s in cell parameters are only used when they are defined by crystal symmetry. An approximate (isotropic) treatment of cell e.s.d.'s is used for estimating e.s.d.'s involving l.s. planes.
Refinement. Refinement of *F*^2^ against ALL reflections. The weighted *R*-factor *wR* and goodness of fit *S* are based on *F*^2^, conventional *R*-factors *R* are based on *F*, with *F* set to zero for negative *F*^2^. The threshold expression of *F*^2^ > σ(*F*^2^) is used only for calculating *R*-factors(gt) *etc*. and is not relevant to the choice of reflections for refinement. *R*-factors based on *F*^2^ are statistically about twice as large as those based on *F*, and *R*- factors based on ALL data will be even larger.

### Fractional atomic coordinates and isotropic or equivalent isotropic displacement parameters (Å^2^)

**Table:**

	*x*	*y*	*z*	*U*_iso_*/*U*_eq_	
Ni1	0.039679 (16)	0.25481 (7)	0.15523 (2)	0.04140 (15)	
Br1	−0.214719 (15)	0.57208 (8)	−0.01235 (2)	0.07427 (19)	
O1	−0.01744 (9)	0.1671 (4)	0.14220 (12)	0.0521 (6)	
O2	0.09732 (8)	0.3432 (4)	0.16657 (11)	0.0461 (6)	
N1	0.04942 (11)	0.6167 (5)	0.09492 (13)	0.0453 (7)	
N2	0.01454 (11)	0.4946 (4)	0.09898 (13)	0.0418 (7)	
N3	0.07198 (11)	0.0131 (5)	0.21834 (14)	0.0447 (7)	
C1	0.25839 (16)	0.9041 (8)	0.1443 (2)	0.0827 (14)	
H1A	0.2839	0.7978	0.1605	0.124*	
H1B	0.2700	1.0277	0.1748	0.124*	
H1C	0.2486	0.9493	0.0983	0.124*	
C2	0.21573 (15)	0.8065 (7)	0.14342 (18)	0.0559 (10)	
C3	0.21831 (14)	0.6056 (7)	0.17226 (19)	0.0601 (11)	
H3	0.2477	0.5306	0.1947	0.072*	
C4	0.17830 (14)	0.5144 (6)	0.16844 (18)	0.0534 (10)	
H4	0.1810	0.3774	0.1875	0.064*	
C5	0.13426 (13)	0.6209 (6)	0.13698 (17)	0.0439 (9)	
C6	0.13174 (15)	0.8249 (7)	0.1090 (2)	0.0594 (10)	
H6	0.1026	0.9018	0.0876	0.071*	
C7	0.17177 (16)	0.9138 (7)	0.1127 (2)	0.0647 (11)	
H7	0.1692	1.0510	0.0938	0.078*	
C8	0.09144 (13)	0.5231 (6)	0.13248 (17)	0.0424 (9)	
C9	−0.03004 (13)	0.5689 (5)	0.06399 (16)	0.0425 (8)	
C10	−0.04099 (14)	0.7722 (6)	0.02212 (18)	0.0537 (10)	
H10A	−0.0467	0.8895	0.0448	0.081*	
H10B	−0.0695	0.7501	−0.0232	0.081*	
H10C	−0.0138	0.8075	0.0179	0.081*	
C11	−0.06813 (12)	0.4555 (6)	0.06651 (16)	0.0405 (8)	
C12	−0.06026 (13)	0.2605 (6)	0.10355 (17)	0.0440 (9)	
C13	−0.09983 (14)	0.1556 (7)	0.09951 (19)	0.0545 (10)	
H13	−0.0948	0.0229	0.1220	0.065*	
C14	−0.14580 (15)	0.2406 (7)	0.0637 (2)	0.0570 (10)	
H14	−0.1718	0.1676	0.0614	0.068*	
C15	−0.15255 (14)	0.4378 (7)	0.03092 (17)	0.0517 (10)	
C16	−0.11546 (14)	0.5416 (6)	0.03128 (17)	0.0479 (9)	
H16	−0.1215	0.6728	0.0077	0.058*	
C17	0.04736 (15)	−0.1465 (6)	0.22590 (18)	0.0509 (9)	
H17	0.0135	−0.1401	0.2007	0.061*	
C18	0.07017 (17)	−0.3209 (7)	0.26964 (19)	0.0587 (11)	
H18	0.0518	−0.4301	0.2733	0.070*	
C19	0.11926 (17)	−0.3323 (7)	0.30699 (19)	0.0645 (12)	
H19	0.1351	−0.4494	0.3364	0.077*	
C20	0.14525 (17)	−0.1681 (8)	0.3008 (2)	0.0700 (12)	
H20	0.1791	−0.1707	0.3264	0.084*	
C21	0.12057 (15)	−0.0002 (7)	0.2563 (2)	0.0626 (11)	
H21	0.1385	0.1105	0.2523	0.075*	

### Atomic displacement parameters (Å^2^)

**Table:**

	*U*^11^	*U*^22^	*U*^33^	*U*^12^	*U*^13^	*U*^23^
Ni1	0.0472 (3)	0.0340 (3)	0.0408 (3)	0.0066 (2)	0.0218 (2)	0.0065 (2)
Br1	0.0558 (3)	0.0908 (4)	0.0771 (3)	0.0253 (2)	0.0359 (2)	0.0117 (3)
O1	0.0495 (15)	0.0417 (15)	0.0595 (15)	0.0057 (13)	0.0252 (13)	0.0160 (12)
O2	0.0519 (15)	0.0379 (14)	0.0463 (13)	0.0045 (12)	0.0246 (12)	0.0109 (12)
N1	0.0529 (19)	0.0368 (19)	0.0454 (16)	0.0034 (15)	0.0256 (15)	0.0046 (14)
N2	0.0534 (19)	0.0298 (16)	0.0438 (16)	0.0051 (14)	0.0269 (14)	0.0033 (13)
N3	0.0533 (19)	0.0390 (19)	0.0411 (16)	0.0073 (15)	0.0246 (15)	0.0047 (13)
C1	0.069 (3)	0.094 (4)	0.083 (3)	−0.022 (3)	0.040 (3)	0.006 (3)
C2	0.058 (3)	0.059 (3)	0.046 (2)	−0.012 (2)	0.025 (2)	−0.0014 (19)
C3	0.050 (2)	0.066 (3)	0.062 (2)	0.003 (2)	0.028 (2)	0.010 (2)
C4	0.060 (3)	0.045 (2)	0.059 (2)	0.005 (2)	0.034 (2)	0.0097 (19)
C5	0.054 (2)	0.037 (2)	0.0426 (19)	0.0020 (18)	0.0266 (18)	−0.0007 (16)
C6	0.060 (3)	0.049 (2)	0.067 (3)	0.006 (2)	0.032 (2)	0.013 (2)
C7	0.075 (3)	0.046 (3)	0.075 (3)	−0.006 (2)	0.041 (2)	0.013 (2)
C8	0.054 (2)	0.034 (2)	0.0400 (19)	0.0012 (18)	0.0247 (17)	0.0000 (16)
C9	0.055 (2)	0.032 (2)	0.0396 (18)	0.0096 (18)	0.0239 (17)	0.0006 (16)
C10	0.062 (3)	0.041 (2)	0.053 (2)	0.0079 (19)	0.028 (2)	0.0117 (18)
C11	0.045 (2)	0.038 (2)	0.0388 (18)	0.0073 (17)	0.0231 (16)	−0.0006 (16)
C12	0.046 (2)	0.043 (2)	0.0408 (19)	0.0072 (18)	0.0215 (17)	0.0007 (17)
C13	0.056 (2)	0.049 (2)	0.065 (2)	0.006 (2)	0.036 (2)	0.010 (2)
C14	0.058 (3)	0.059 (3)	0.064 (2)	0.002 (2)	0.040 (2)	−0.001 (2)
C15	0.053 (2)	0.061 (3)	0.045 (2)	0.012 (2)	0.0281 (18)	−0.0001 (19)
C16	0.055 (2)	0.044 (2)	0.045 (2)	0.0145 (19)	0.0264 (18)	0.0059 (17)
C17	0.061 (2)	0.039 (2)	0.050 (2)	0.005 (2)	0.0271 (19)	0.0058 (18)
C18	0.085 (3)	0.042 (2)	0.054 (2)	0.006 (2)	0.041 (2)	0.0081 (19)
C19	0.090 (3)	0.054 (3)	0.053 (2)	0.029 (3)	0.040 (3)	0.016 (2)
C20	0.067 (3)	0.069 (3)	0.073 (3)	0.029 (3)	0.036 (2)	0.030 (2)
C21	0.059 (3)	0.063 (3)	0.069 (3)	0.016 (2)	0.036 (2)	0.024 (2)

### Geometric parameters (Å, °)

**Table:**

Ni1—O1	1.794 (3)	C6—H6	0.9300
Ni1—O2	1.826 (3)	C7—H7	0.9300
Ni1—N2	1.835 (3)	C9—C11	1.444 (5)
Ni1—N3	1.941 (3)	C9—C10	1.495 (5)
Br1—C15	1.897 (4)	C10—H10A	0.9600
O1—C12	1.315 (4)	C10—H10B	0.9600
O2—C8	1.305 (4)	C10—H10C	0.9600
N1—C8	1.295 (4)	C11—C16	1.402 (5)
N1—N2	1.399 (4)	C11—C12	1.408 (5)
N2—C9	1.306 (4)	C12—C13	1.393 (5)
N3—C17	1.331 (5)	C13—C14	1.365 (5)
N3—C21	1.336 (5)	C13—H13	0.9300
C1—C2	1.496 (5)	C14—C15	1.378 (5)
C1—H1A	0.9600	C14—H14	0.9300
C1—H1B	0.9600	C15—C16	1.356 (5)
C1—H1C	0.9600	C16—H16	0.9300
C2—C7	1.376 (6)	C17—C18	1.380 (5)
C2—C3	1.380 (5)	C17—H17	0.9300
C3—C4	1.371 (5)	C18—C19	1.349 (6)
C3—H3	0.9300	C18—H18	0.9300
C4—C5	1.375 (5)	C19—C20	1.368 (6)
C4—H4	0.9300	C19—H19	0.9300
C5—C6	1.388 (5)	C20—C21	1.367 (5)
C5—C8	1.464 (5)	C20—H20	0.9300
C6—C7	1.368 (5)	C21—H21	0.9300
			
O1—Ni1—O2	178.81 (10)	N2—C9—C11	119.9 (3)
O1—Ni1—N2	95.14 (12)	N2—C9—C10	119.5 (3)
O2—Ni1—N2	84.33 (12)	C11—C9—C10	120.7 (3)
O1—Ni1—N3	89.75 (12)	C9—C10—H10A	109.5
O2—Ni1—N3	90.82 (12)	C9—C10—H10B	109.5
N2—Ni1—N3	174.87 (13)	H10A—C10—H10B	109.5
C12—O1—Ni1	127.4 (2)	C9—C10—H10C	109.5
C8—O2—Ni1	110.6 (2)	H10A—C10—H10C	109.5
C8—N1—N2	108.7 (3)	H10B—C10—H10C	109.5
C9—N2—N1	116.2 (3)	C16—C11—C12	117.3 (3)
C9—N2—Ni1	130.1 (3)	C16—C11—C9	119.5 (3)
N1—N2—Ni1	113.7 (2)	C12—C11—C9	123.2 (3)
C17—N3—C21	116.8 (3)	O1—C12—C13	116.6 (3)
C17—N3—Ni1	122.3 (3)	O1—C12—C11	124.3 (3)
C21—N3—Ni1	120.8 (3)	C13—C12—C11	119.1 (3)
C2—C1—H1A	109.5	C14—C13—C12	122.3 (4)
C2—C1—H1B	109.5	C14—C13—H13	118.8
H1A—C1—H1B	109.5	C12—C13—H13	118.8
C2—C1—H1C	109.5	C13—C14—C15	118.0 (4)
H1A—C1—H1C	109.5	C13—C14—H14	121.0
H1B—C1—H1C	109.5	C15—C14—H14	121.0
C7—C2—C3	117.1 (4)	C16—C15—C14	121.6 (4)
C7—C2—C1	120.9 (4)	C16—C15—Br1	119.4 (3)
C3—C2—C1	122.0 (4)	C14—C15—Br1	118.9 (3)
C4—C3—C2	121.3 (4)	C15—C16—C11	121.4 (4)
C4—C3—H3	119.4	C15—C16—H16	119.3
C2—C3—H3	119.4	C11—C16—H16	119.3
C3—C4—C5	121.5 (4)	N3—C17—C18	122.5 (4)
C3—C4—H4	119.3	N3—C17—H17	118.7
C5—C4—H4	119.3	C18—C17—H17	118.7
C4—C5—C6	117.4 (3)	C19—C18—C17	119.6 (4)
C4—C5—C8	121.5 (3)	C19—C18—H18	120.2
C6—C5—C8	121.0 (3)	C17—C18—H18	120.2
C7—C6—C5	120.6 (4)	C18—C19—C20	118.7 (4)
C7—C6—H6	119.7	C18—C19—H19	120.6
C5—C6—H6	119.7	C20—C19—H19	120.6
C6—C7—C2	122.1 (4)	C21—C20—C19	118.9 (4)
C6—C7—H7	119.0	C21—C20—H20	120.6
C2—C7—H7	119.0	C19—C20—H20	120.6
N1—C8—O2	122.6 (3)	N3—C21—C20	123.4 (4)
N1—C8—C5	119.2 (3)	N3—C21—H21	118.3
O2—C8—C5	118.2 (3)	C20—C21—H21	118.3
			
N2—Ni1—O1—C12	0.0 (3)	N1—N2—C9—C11	−178.7 (3)
N3—Ni1—O1—C12	178.4 (3)	Ni1—N2—C9—C11	−0.2 (5)
N2—Ni1—O2—C8	1.6 (2)	N1—N2—C9—C10	0.8 (4)
N3—Ni1—O2—C8	−176.7 (2)	Ni1—N2—C9—C10	179.3 (2)
C8—N1—N2—C9	179.6 (3)	N2—C9—C11—C16	177.2 (3)
C8—N1—N2—Ni1	0.9 (3)	C10—C9—C11—C16	−2.3 (5)
O1—Ni1—N2—C9	1.2 (3)	N2—C9—C11—C12	−2.2 (5)
O2—Ni1—N2—C9	−179.9 (3)	C10—C9—C11—C12	178.3 (3)
O1—Ni1—N2—N1	179.6 (2)	Ni1—O1—C12—C13	177.4 (2)
O2—Ni1—N2—N1	−1.4 (2)	Ni1—O1—C12—C11	−2.2 (5)
O1—Ni1—N3—C17	9.5 (3)	C16—C11—C12—O1	−175.9 (3)
O2—Ni1—N3—C17	−169.5 (3)	C9—C11—C12—O1	3.5 (5)
O1—Ni1—N3—C21	−170.7 (3)	C16—C11—C12—C13	4.5 (5)
O2—Ni1—N3—C21	10.3 (3)	C9—C11—C12—C13	−176.1 (3)
C7—C2—C3—C4	2.0 (6)	O1—C12—C13—C14	177.0 (3)
C1—C2—C3—C4	−177.2 (4)	C11—C12—C13—C14	−3.4 (5)
C2—C3—C4—C5	−1.3 (6)	C12—C13—C14—C15	−0.3 (6)
C3—C4—C5—C6	0.1 (5)	C13—C14—C15—C16	2.9 (5)
C3—C4—C5—C8	179.6 (3)	C13—C14—C15—Br1	−174.5 (3)
C4—C5—C6—C7	0.4 (5)	C14—C15—C16—C11	−1.6 (5)
C8—C5—C6—C7	−179.2 (3)	Br1—C15—C16—C11	175.8 (2)
C5—C6—C7—C2	0.4 (6)	C12—C11—C16—C15	−2.1 (5)
C3—C2—C7—C6	−1.5 (6)	C9—C11—C16—C15	178.4 (3)
C1—C2—C7—C6	177.7 (4)	C21—N3—C17—C18	−1.2 (5)
N2—N1—C8—O2	0.6 (4)	Ni1—N3—C17—C18	178.6 (3)
N2—N1—C8—C5	−179.8 (3)	N3—C17—C18—C19	0.5 (6)
Ni1—O2—C8—N1	−1.7 (4)	C17—C18—C19—C20	0.6 (6)
Ni1—O2—C8—C5	178.6 (2)	C18—C19—C20—C21	−0.9 (6)
C4—C5—C8—N1	−171.3 (3)	C17—N3—C21—C20	0.8 (6)
C6—C5—C8—N1	8.2 (5)	Ni1—N3—C21—C20	−179.0 (3)
C4—C5—C8—O2	8.3 (5)	C19—C20—C21—N3	0.2 (7)
C6—C5—C8—O2	−172.2 (3)		
